# Carrot Juice Intake Affects the Cytokine and Chemokine Response in Human Blood after Ex Vivo Lipopolysaccharide-Induced Inflammation

**DOI:** 10.3390/nu15235002

**Published:** 2023-12-02

**Authors:** Morten Kobaek-Larsen, Ulrik Deding, Issam Al-Najami, Bettina Hjelm Clausen, Lars Porskjær Christensen

**Affiliations:** 1 Department of Surgery, Odense University Hospital, DK-5000 Odense C, Denmark; mkobaek@hotmail.com (M.K.-L.); ulrik.deding@rsyd.dk (U.D.); issam.al-najami@rsyd.dk (I.A.-N.); 2 Department of Clinical Research, University of Southern Denmark, DK-5230 Odense M, Denmark; 3 Department of Neurobiology Research, Institute of Molecular Medicine, University of Southern Denmark, DK-5000 Odense C, Denmark; bclausen@health.sdu.dk; 4 Department of Physics, Chemistry and Pharmacy, University of Southern Denmark, DK-5230 Odense M, Denmark

**Keywords:** carrots, inflammation, lipopolysaccharide, ex vivo, cytokines, falcarinol, falcarindiol

## Abstract

In vitro and animal studies have shown that carrot juice containing bioactive natural products, such as falcarinol (FaOH) and falcarindiol (FaDOH), can affect inflammation. The present study was designed to test whether oral intake of carrot juice containing the bioactive acetylenic oxylipins FaOH and FaDOH affects mediators of acute inflammation or the innate immune response in human blood. Carrot juice (500 mL) was administered orally to healthy volunteers, and blood samples were drawn before and 1 h after juice intake. Next, the blood samples were split in two, and one sample was stimulated ex vivo with lipopolysaccharide (LPS) and incubated at 37 °C for 24 h. The concentrations of 44 inflammatory cytokines and chemokines were examined using multiplex electrochemiluminescence analysis. In blood samples not stimulated with LPS, a significant increase in IL-15 was measured 1 h after carrot juice intake. Cytokines like IFN-ɣ, IL-12/IL-23(p40), IL-23, IL-17A, IL-17B, IL-17D, and IL-22 were significantly increased in LPS-stimulated blood samples after carrot juice intake. The upregulation of the immunostimulating cytokines belonging to the IL-23/IL-17 Th17 axis suggests that carrot juice intake could benefit diseases where inflammation plays a role, like in the early stages of diabetes or cancers.

## 1. Introduction

Chronic and persistent inflammation makes individuals susceptible to the development of many diseases, including diabetes, cancer, and cardiovascular diseases. It has previously been demonstrated that carrots (*Daucus carota* L.) can contribute to functional tissue recovery in these diseases and that the responsible compounds in carrots are the C_17_ acetylenic oxylipins falcarinol (FaOH) and falcarindiol (FaDOH) [1,2,3,4,5,6,7,8,9,10]. FaOH and FaDOH have, in numerous in vitro and/or in vivo studies, shown anti-inflammatory effects on common inflammatory biomarkers such as cyclooxygenase (COX)-1 and -2, lipoxygenase-5, -12, and -15, NF-κβ, TNFα, and interleukin (IL)-6, as well as demonstrated antiplatelet-aggregatory, anti-diabetic, antiproliferative, and antitumor activity [2,4,6,7,11,12,13,14,15,16,17,18,19,20,21,22,23]. Furthermore, it has been demonstrated that long-term intake of FaOH and FaDOH downregulates the gene expression of cyclooxygenase-2 (COX-2) as well as the inflammatory cytokines interleukin (IL)-6 and tumor necrosis factor α (TNFα) in the colonic epithelium in a rat model of colorectal cancer (CRC) [4]. In the same rat study, the downregulation of nuclear factor kappa-light-chain-enhancer of activated B cells (NF-κβ), which is responsible for the production of cytokines and COX-2, was also observed, whereas no effect on the gene expression of COX-1 was detected [4].

COX-2 levels are low in healthy tissue but are rapidly induced as an early response to growth factors, cytokines, and tumor promoters associated with inflammation, abnormal cell proliferation, angiogenesis, and metastasis [24]. A connection between CRC and COX-2 overexpression has been established in accordance with the chemopreventive effect of FaOH and FaDOH in a rat model for CRC [24,25], and just recently, we demonstrated that intake of carrot juice in healthy human individuals reduced the secretion of inflammatory cytokines like IL-1α and IL-16, not of COX-2, IL-6, and TNFα, following LPS stimulation ex vivo, which are all known players in cancer development [1]. 

FaOH and FaDOH are, besides carrots, also present in many other food plants of the Apiaceae family, such as celeriac (*Apium graveolens* L. var. *rapaceum*), celery (*A. graveolens* L. var. *dulce*), fennel (*Foeniculum vulgare* Mill.), parsnip (*Pastinaca sativa* L.), lovage root (*Levisticum officinale* W. D. J. Koch), and turnip-rooted parsley (*Petroselinum crispum* Mill. var. *tuberosum*), as well as in many medicinal plants of the Araliaceae family like the famous ginseng root (*Panax ginseng* C. A. Meyer) [2,16,18]. Carrot is, however, the most widely consumed vegetable, thus contributing to most of the dietary intake of FaOH and FaDOH [12,16,26]. The content of FaOH and FaDOH depends on carrot cultivars and growing conditions and may vary between 40 to 600 mg/kg dry weight (DW) [16,26]. However, a serum level in humans of around 4 ng/mL blood can still be reached by eating 300 g of commercially available carrots [16,27]. This is within the range where an effect on the growth of human cells in vitro is observed [12,13,14,15,16,28]. Based on the systematic investigation of FaOH and FaDOH and their interaction with human cancer cells and enzyme systems, these compounds appear to play a prophylactic role in some vegetable foods and medicinal plants [2,16]. These effects of FaOH and FaDOH are most likely due to their triple bond functionality, which transforms them into highly alkylating compounds. The alkylating properties of FaOH and FaDOH make them reactive towards proteins and other biomolecules, whereby they are able to induce the formation of anti-inflammatory and cytoprotective phase 2 enzymes via activation of the Kelch-like ECH-associated protein 1 (Keap1)-nuclear factor erythroid 2-related factor 2 (Nrf2)-antioxidant response elements (ARE) signaling pathway, the inhibition of proinflammatory peptides and proteins, and/or the induction of endoplasmic reticulum (ER) stress [2,29,30,31], and thus can explain their prophylactic effects on inflammatory-related diseases and in particular cancer. 

The anti-inflammatory and antiproliferative effects of carrots and their bioactive constituents indicate an effect on the innate immune system. However, it is unclear how carrots and their bioactive metabolites exert their immune-modulating effects in humans. In this study, we have comprehensively investigated the effect of carrot juice intake on cytokine and chemokine secretion under healthy and inflamed conditions ex vivo to gain a greater insight into the immune-modulating effects of carrots. Cytokines and chemokines are important multifunctional mediators of cell behavior and cell-to-cell communication and play an essential role in the innate immune system. The functions of cytokines and chemokines vary from being stimulatory and inhibitory to migratory, and how they affect the cells in the environment is frequently also dependent on the presence or absence of other cytokines, and thus the inflammatory and health status of individuals [32,33]. The present study, therefore, provides new important knowledge on the immune-modulating effects of carrots and their potential health-promoting effects. 

## 2. Materials and Methods

### 2.1. Study Subjects 

Fourteen healthy volunteers (9 females and 5 males, aged 20–55 years) underwent blood sampling before and 1 h after carrot juice intake. Consent to participate in the study was obtained from each participant. Subjects took no NSAIDs or carrots/carrot products 48 h preceding the study. All samples have been part of a study on COXs and a few selected cytokines, as previously described [1]. This study was approved by the Regional Health Research Ethics Committee (S-20210071).

### 2.2. Carrot Juice 

Carrot materials (cv. Night Bird F1 hybrid) were grown organically at DanRoots A/S (Bjerringbro, Denmark). Tops and bottoms were removed from fresh, washed carrots, which were then shredded, freeze-dried, and prepared into powder (European Freeze-Dry, Kirke Hyllinge, Denmark). The powder was packed in sealed aluminum foil pouches and stored at −30 °C until use.

The carrot juice was prepared in a 600 mL plastic shaker on the same day as the tests were conducted. The juice contained 30 g of freeze-dried carrot powder and 500 mL of tap water, giving a maximum concentration of FaOH in circulation 1 h after juice intake in humans [27].

### 2.3. Blood Sampling 

For each individual, two times 4 mL aliquots of peripheral venous blood samples were transferred into glass tubes containing 10 IU of sodium heparin (plasma samples) before oral intake of carrot juice. This sampling was repeated 1 h after oral intake of carrot juice. 

### 2.4. LPS Mediated Stimulation of Leucocytes/Monocytes in Human Blood 

**Figure 1:**
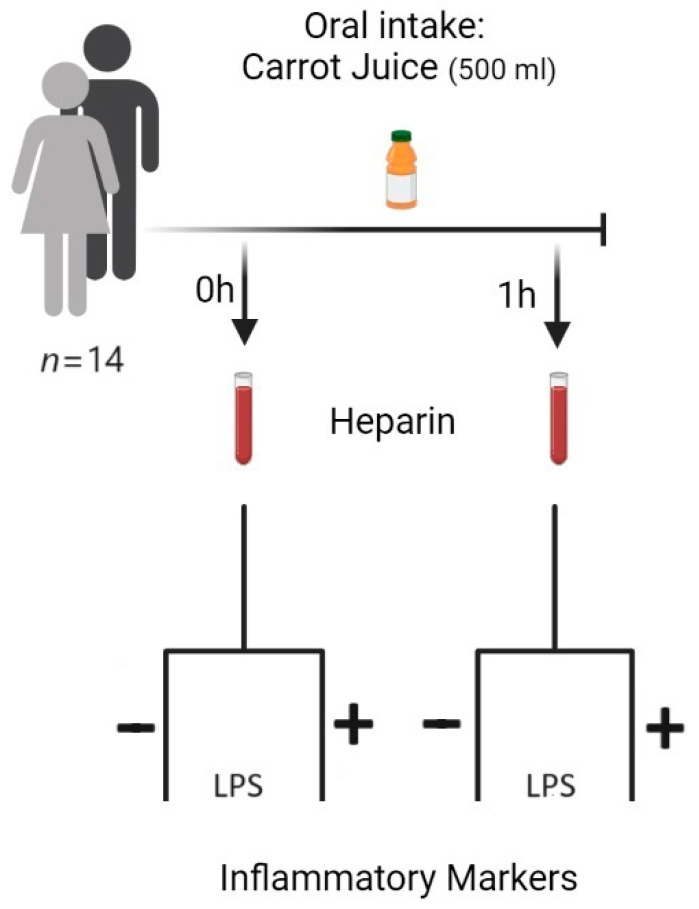
Illustration of the experimental setup used in the study. The participants (*n* = 14) consist of 9 females and 5 males. The volunteers were used as their own control as the native blood samples from the same volunteers were analyzed before and after the intake of carrot juice. The blood samples taken before and 1 h after intake of carrot juice were analyzed in the presence and absence of LPS. The volunteers were not fasting before the carrot intake but were restricted to no intake of carrots or carrot products as well as NSAID medication 48 h before the study. Created with BioRender.com.

An amount of 4 mL aliquots of human whole blood containing 10 IU of sodium heparin was incubated either in the absence or presence of LPS (10 µg/mL) (*E. coli* O111:B4, MERCK) for 0 to 24 h, at 37 °C. Plasma was separated by centrifugation (10 min at 2000 rpm) and kept at −30 °C until assayed for inflammatory markers [34,35] (Figure 1). 

### 2.5. Analysis of Cytokines and Chemokines in Plasma 

Changes in the concentration of 44 human pro-inflammatory and anti-inflammatory cytokines in plasma were analyzed in duplicate using the V-Plex Plus human cytokine kits (Mesoscale, Rockville, MD, USA, Catalog no. K15249D) in accordance with manufacturer’s guidelines. The plates were read using the MSD QuickPlex (SQ120) Plate Reader (Mesoscale Discovery), and the data were analyzed using the MSD Discovery Workbench software, as detailed in [36]. An overview of the multi-plex plate containing analytes for the analysis of pro-inflammatory cytokines, pro-inflammatory chemokines, and anti-inflammatory cytokines is shown in Supplemental Tables S1 and S2.

### 2.6. Protein–Protein Interaction Network Analysis

The STRING 11 online tool (https://version-11-0.string-db.org/ (accessed on 11 October 2023) [37] was used to analyze protein–protein interaction networks of the significantly altered cytokines in LPS-stimulated blood samples before and after intake of carrot juice. The protein-interaction network graph was based on data on protein–protein interactions including binding, activation, inhibition, posttranslational modification, transcriptional regulation, etc. 

### 2.7. Statistical Analysis

Wilcoxon signed-rank tests were performed to compare the excretion of each biomarker, respectively, at baseline and 1 h after carrot juice intake. This was repeated for samples subjected to LPS stimulation. The level of statistical significance was set at 5% (*p* < 0.05). All data management and statistical analyses were performed in SAS software version 9.4 (SAS Institute Inc. SAS 9.4. Cary, NC, USA) and R statistical software package version 3.6.1 (R Core Team, Vienna, Austria).

## 3. Results

### 3.1. Carrot Juice Intake Affects Cytokine and Chemokine Concentrations in Human Plasma 

At baseline, plasma was analyzed before and after intake of carrot juice. Most analytes showed no change in plasma concentration between baseline and 1 h after carrot juice intake except IL-15, which significantly increased (*p* = 0.0105) in plasma (Table 1).

In contrast, carrot juice intake affected both pro- and anti-inflammatory cytokines in LPS-stimulated plasma samples 1 h after juice intake compared to no juice intake (Table 1). Levels of IFN-γ, IL-17A, IL-17B, IL-17D, IL-22, IL-23, and IL-12/IL-23p40 were significantly changed following carrot juice intake and LPS-stimulation (Table 1) in plasma samples with a peak concentration of FaOH [27]. However, we did not observe any gender differences concerning the production of cytokines. The results of all analyzed cytokines and chemokines are shown in Supplemental Table S2.

### 3.2. Effect of Carrot Juice Intake in LPS-Stimulated Human Plasma

**Figure 2:**
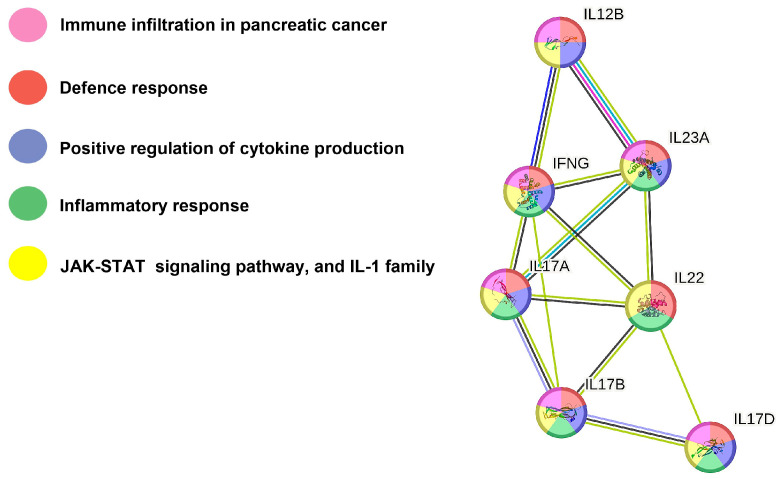
Illustration of the STRING analysis showing the interplay of the cytokines IL-23, IL-22, IL-17A, IL-17B, IL-17D, and IFN-γ. STRING interaction network depicting proteins involved in the defense response (red nodes). Positive regulation of cytokine production (blue nodes) and inflammatory response (green nodes). Pink nodes represent proteins involved in immune infiltration in pancreatic cancer. Yellow nodes indicate proteins affecting the JAK-STAT pathway and the IL-1 family.

To reveal the effect of carrot juice intake on stimulated cytokine synthesis and release, the TLR4 agonist LPS was added to whole blood drawn before and after carrot juice intake. Plasma was separated after incubation for 24 h. Addition of LPS increased plasma concentration of most cytokines above baseline in the present investigation with the largest fold-increase for GS-CSF (528-fold), IL-10 (44-fold), MIP-1α (38-fold), and IL-8 (23-fold), while IL-17A GenB, IL-15, IL-17D, IL-21, IL-31, VEGF-A, Eotaxin-1, MCP-4, and MDC were not increased or only to a minor extent (Table 1 and Table S2). Comparison of the paired samples with LPS before and after carrot juice showed that the addition of LPS to the blood samples drawn before and 1 h after carrot juice intake resulted in a significantly increased secretion of the pro-inflammatory cytokines IFN-γ (*p* = 0.0166), IL-17A (*p* = 0.0023), IL-17B (*p* = 0.0134), IL-17D (*p* = 0.0085), and IL-23 (*p* = 0.0002), and the anti-inflammatory cytokines IL-12/IL-23p40 (*p* = 0.0245) and IL-22 (*p* = 0.0215) in human plasma samples. However, we did not observe any gender differences concerning the production of cytokines. STRING network analysis was performed to reveal the protein–protein interaction network between these identified cytokines (Figure 2). Based on interaction evidence [37], the cytokines are involved in immune infiltration in cancers, defense responses, positive regulation of cytokine production, and the inflammatory response; all the identified cytokines also stimulate the Janus kinase (JAK)-signal transducer and activator of transcription (STAT) signaling pathway (Figure 2). 

## 4. Discussion

Carrots and their bioactive constituents FaOH and FaDOH have, in numerous investigations, been shown to be potent inhibitors of inflammation and to inhibit the proliferation of mammalian cells, including cancer cells [2,4,6,10,11,12,13,14,15,16,17,18,19,20]. In addition, FaOH and FaDOH have shown a synergistic inhibitory effect on human intestinal cells of normal and cancer origin demonstrating an enhanced antiproliferative effect in vitro in different ratios [12]. The ratio 1:1 of FaOH and FaDOH was used to demonstrate the anti-inflammatory and anticancer effect of FaOH and FaDOH in the rat model of CRC described in the introduction [4]. In this context, the activation of the Keap1-Nrf2 pathway by FaOH and FaDOH may constitute an important mechanism of action for their health-promoting effects. Nrf2 has been implicated in the regulation of the oxidative stress response and inflammatory responses and as an important regulator of innate immunity through the formation of pro- and anti-inflammatory cytokines [38,39,40]. The implication of Nrf2 in controlling the immune response has been shown to be due to direct or indirect interaction with important innate immune components, including the toll-like receptors–NF-kB pathway, inflammasome signaling, and the type-I interferon response indicating an essential role for Nrf2 in diseases related to inflammation, cancer, and microbial infections [38,39,40]. In resting cells, Nrf2 is bound to Keap1; however, upon exposure to various stimuli, including reactive oxygen species, nitrogen species, and electrophilic molecules such as FaOH and FaDOH, Nrf2 is activated and released from the Keap1 complex and translocated to the nucleus to activate its target genes recruiting immune cells to the site of infection through the formation of cytokines. 

Several studies have demonstrated that Nrf2 contributes to the anti-inflammatory process and that there is a connection between Nrf2 and signaling pathways associated with the inflammatory response [40,41,42]. STRING analysis of the cytokines affected by the intake of carrot juice in the used ex vivo model showed that besides interaction with the JAK-STAT signaling pathway, carrots also appear to have an impact on immune infiltration of cancers, the defense and inflammatory response in the humane body, as well as the positive regulation of the cytokine production (Figure 2). The JAK-STAT signaling pathway is central to extracellular cytokine-activated receptor-mediated signal transduction, which is involved in cellular proliferation, differentiation, inflammation, and immune homeostasis [43]. Thus, the STRING analysis indicates that the intake of carrots has health-promoting effects, which may be linked to their immunomodulatory and immunostimulating effect.

The ex vivo setup used in this study was a human model of acute inflammation and immunostimulation to investigate the effect of intake of carrots on monocytes and macrophages in blood samples from healthy volunteers with and without immunostimulation by LPS [1]. Thus, this model system represents the effect of intake of carrots on parts of the innate immune system and its effects on acute inflammatory response. Acute inflammation caused by LPS resulted in increased excretion of the IL-12/IL-23p40, IL-23, and IL-17 families (IL-17A, B, and D) of cytokines and IL-22, which were further increased 1 h after intake of carrots (Table 1), whereas it has previously been shown that the excretion of IL-1α and IL-16 decreased in this ex vivo model [1]. 

IL-23 is an inflammatory cytokine, which has been shown to be a key cytokine for T helper (Th) cell maintenance and expansion [44,45]. IL-23 is mainly secreted by activated macrophages or monocytes in the ex vivo blood samples. The cytokine IL-23 stimulates the Th17 cells to secrete IL-17 and IL-22 cytokines. The increased IL-23 level is, therefore, in accordance with the observed increase in the level of IL-17 cytokines and IL-22 in the blood samples after stimulation with LPS and 1 h after intake of carrot juice (Table 1). The IL-17 family of cytokines is composed of six members named IL-17A (commonly known as IL-17), IL-17B, IL-17C, IL-17D, IL-17E (also known as IL-25), and IL-17F with different sequence homology and functions that are important players in host defense responses, inflammation, and cancer development [46,47,48]. The IL-17 cytokines exert their activities through binding to IL-17 receptors (IL-17RA to IL-17RE) that function as homo- or heterodimeric complexes leading to the production of other cytokines and chemokines. IL-17A is the prototypic member of the IL-17 family and was the first member of the IL-17 family to be discovered and targeted in the clinic. IL-17A has received much attention for its pro-inflammatory role in autoimmune disease, but accumulating evidence indicates that IL-17A has important context- and tissue-dependent roles in maintaining health during response to injury, physiological stress, and infection [46,47,48]. IL-17A derived from the innate and adaptive immune system is essential for modulating the interplay between commensal microbes and epithelial cells at our borders (i.e., skin and mucosae) at different phases and locations of infection, protecting us from microbial invaders, and thus preserving mucosal and skin integrity [46,47,48]. However, IL-17A overproduction has been associated with chronic inflammatory disorders, autoimmune diseases, and cancers [49,50,51]. Therefore, IL-17A possesses a dual role that, under some conditions, will protect from diseases, while under other conditions, it could harm the human body [52,53]. In one case report, anti-IL-17A treatment was beneficial for preventing the development of an autoimmune disease in a patient, but at the same time, it caused the loss of an antitumor response in the same patient who also suffered from CRC [54]. Furthermore, the antitumor effect has been demonstrated in mice models of cancer [55]. Whether the outcomes of IL-17A signaling are beneficial or harmful depends on the amount of IL-17A produced and how IL-17A signals are received and transmitted within the responding cell. The related IL-17B share the common interleukin receptor IL17-RB with IL-25, and they are expressed in several peripheral tissues and immune tissues, including colon epithelial cells [56]. However, IL-17B and IL-25 appear to have opposite functions in colon inflammation. In acute colonic inflammation, both cytokines have been shown to be upregulated, where IL-25 has a pathogenic role by promoting the production of the pro-inflammatory mediator IL-6, whereas IL-17B has been shown to perform an anti-inflammatory role by blocking IL-25 signaling in acute inflammation [47,56]. In this study, IL-6 was not upregulated, which could be indicative of a beneficial immune response of IL-17B. Although IL-17B promotes cell survival, proliferation, and migration, when measured alone, it relates to poor prognosis in patients with different types of cancer [47]. 

IL-17D is expressed in a wide variety of healthy tissues but is one of the least understood members of the IL-17 family [47]. Stimulation of IL-17D in endothelial cells has been shown to induce classical pro-inflammatory cytokine responses such as IL-6, IL-8, and GM-CSF [57], although increased levels of IL-17D in the present study did not result in increased levels of these pro-inflammatory cytokines. In addition, IL-17D has been found to increase in tumors and during viral infections where Nrf2-mediated IL-17D expression plays an important role in the antitumor as well as the antiviral response through the regulation of innate immune cell recruitment [41,47]. Thus, the increased levels of IL-17D observed in the present study may contribute to an anti-inflammatory effect.

IL-22 primarily targets epithelial and stroma cells to induce innate host defense mechanisms that control the invasion of extracellular pathogens. In addition, IL-22 can enhance tissue regeneration and wound healing and thus may provide therapeutic potential in diseases associated with tissue damage [58]. In addition, IL-22 promotes the expression of various chemokines from epithelial cells, which likely helps to recruit the leukocytes to the site of inflammation and/or pathogens [59]. Colorectal cancer and other lifestyle diseases are often initiated during inflammatory conditions and changes in the microbiota. Even a small amount of blood in the stool may be a result of microinflammation and microbiota changes, and blood in the stool can predict the cause of mortality, not only for CRC [60,61]. The increased secretion of IL-23, IL-22, and IL-17 cytokines observed in the ex vivo model by the intake of carrot juice indicates that they will further activate the innate immune response in the event that an acute inflammatory condition occurs, which will result in a reduction of acute microinflammation in the human body. This may help the body to create homeostasis in the microbiota, preventing chronic inflammation and the development of serious diseases because an imbalance in the concentration of IL-23, IL-22, and IL-17 cytokines observed in chronic inflammation is associated with autoimmune diseases and cancer [53,62,63,64].

IL-16 has been shown to exert a strong chemoattractant activity on Th cells, activate the expression and production of proinflammatory cytokines such as IL-1β, IL-6, IL-15, and TNF-α in human monocytes, and play a key role in inflammation, including inflammatory bowel disease, and the progression of certain types of cancers [65,66,67]. The reduced level of IL-16 in the ex vivo setup as previously described [1], and the fact that IL-1β, IL-6, IL-15, and TNF-α are not affected in the blood samples after stimulation with LPS (Table 1 and Table S2 and [1]), indicates that the intake of carrot juice exerts an anti-inflammatory role in relation to IL-16 production and thus may contribute to control the immune response in acute inflammation. The same is true for the reduced level of IL-1α in the ex vivo setup [1] because IL-1α is a damage-associated molecular pattern-induced cytokine that evokes many inflammatory reactions via the IL-1 receptor type 1 [68].

In addition, it was demonstrated in this study that the intake of carrots stimulates the secretion of IL-15 under normal conditions and not in response to inflammation (Table 1). IL-15 is a cytokine with a broad range of biological effects on the innate and adaptive immune system that plays a central role in the inflammatory and protective immune response against pathogens [69,70,71]. This immunostimulating effect of IL-15 stimulates the immune response in general and may prepare the human body for the initiation of inflammation, and this may prevent the development of lifestyle diseases like CRC [72].

Finally, it was demonstrated that IFN-γ was upregulated during the LPS stimulation and after intake of carrots (Table 1). IFN-γ is originally thought to affect viral replication; however, immunostimulating effects are also observed by this cytokine in several complex processes in diseases and health [73,74]. Therefore, the increased IFN-γ secretion observed in the acute inflammation model confirms the immunostimulating effects of carrots. 

The ex vivo model system used in this study to determine the effect of carrot juice on the profile of cytokines and chemokines in the blood circulation has, however, some limitations. First, the measurements reflect a specific point of time before and after the intake of carrot juice (1 h) and, furthermore, acute inflammation due to LPS stimulation of the blood samples. Secondly, the effect of carrot juice on cytokines and chemokines is not determined during pathological processes and specific tissues but is limited to the blood circulation. Finally, the interplay between cytokines and chemokines in tissue is very complex and may be different from that observed in the blood circulation. Consequently, further research is warranted to elucidate the underlying mechanisms for the health-promoting effects of carrot product consumption related to inflammation and immune responses and to validate these observations in long-term clinical trials. Further, such clinical trials should comprise larger and more diverse populations including gender, age, etc., than used in the present study and include the evaluation of the effects on inflammation and immune responses on different cell types and tissues. 

## 5. Conclusions

In the present study, the intake of carrot juice reduced inflammatory cytokines such as IL-15 but upregulated immunostimulating cytokines in the IL-23/IL-17 Th17 axis after induction of acute inflammation by LPS ex vivo. Whether these responses may be harmful or beneficial in the long term is not clarified by the present study. Intake of carrots has acute effects on reactivity in parts of the innate immune system, and the bioactive compounds in carrots that can explain these effects are most likely acetylenic oxylipins such as FaOH and FaDOH due to their ability to activate the pathways of Keap1-Nrf2 and JAK-STAT. 

## Figures and Tables

**Table 1:** Overview of pro- and anti-inflammatory cytokines measured in the plasma samples and affected by carrot juice intake. NSE = no significant effect. Mean concentrations of cytokines pg/mL ± standard deviation (*n* = 14).

Biomarker	Plasma from Whole Blood before and after Intake of Carrot Juice			Plasma from LPS (10 μg/mL, 24 h)-Stimulated Whole Blood before and after Intake of Carrot Juice		
Time of Sampling	0 h	1 h		0 h	1 h	
	Mean (pg/mL)	Mean (pg/mL)	*p*-Value	Mean (pg/mL)	Mean (pg/mL)	*p*-Value
Pro-Inflammatory Cytokines						
IFN-γ	26.5 ± 16.6	26.9 ± 16.5	NSE	252.7 ± 235.7	337.4 ± 278.2	0.0166
IL-15	1.5 ± 0.35	1.8 ± 0.47	0.0105	2.0 ± 0.68	2.2 ± 0.83	NSE
IL-17A	7.3 ± 2.1	7.2 ± 2.7	NSE	96.6 ± 69.0	149.9 ± 83.9	0.0023
IL-17B	6.3 ± 7.6	7.2 ± 7.4	NSE	15.1 ± 9.7	19.2 ± 11.5	0.0134
IL-17D	39.4 ± 18.0	45.3 ± 18.6	NSE	48.9 ± 27.7	62.3 ± 24.8	0.0085
IL-23	2.5 ± 1.9	4.5 ± 4.7	NSE	135.9 ± 184.4	207.9 ± 204.2	0.0002
Anti-Inflammatory Cytokines						
IL-12/IL-23p40	133.3 ± 41.9	141.3 ± 32.3	NSE	3011 ± 2395	4131 ± 2594	0.0245
IL-22	4.4 ± 2.5	4.9 ± 2.6	NSE	8.0 ± 4.4	11.2 ± 8.2	0.0215

## Data Availability

Data are contained within the article and Supplementary Materials.

## Supplementary Materials

The following supporting information can be downloaded at: https://www.mdpi.com/article/10.3390/nu15235002/s1, Tables S1: Overview of the multi-plex plate containing analytes for the analysis of proinflammatory cytokines, pro-inflammatory chemokines, and anti-inflammatory cytokines; Table S2: Effect of carrot juice intake on pro- and inflammatory biomarkers analyzed in an ex *vivo assay* using whole blood from healthy donors with and without addition of LPS. Time of blood sampling 0 h is before consumption of carrot juice and 1 h is after consumption of carrot juice (peak concentration of FaOH in the circulation [27]. NSE = no significant effect. Mean concentrations (pg/mL) of biomarkers measured in the plasma sample are listed for the study (n = 14).
