# Supplementary material for: Carrot Juice Intake Affects the Cytokine and Chemokine Response in Human Blood after Ex Vivo Lipopolysaccharide-Induced Inflammation
Source: Nutrients. 2023 Dec 2;15(23):5002. doi: 10.3390/nu15235002 (PMC10707883; doi:10.3390/nu15235002)
Supplement: Supplementary file 1 [file nutrients-15-05002-s001.zip › nutrients-2731513-supplementary.pdf]

Supplemental Table S1. Overview of the multi-plex plate containing analytes for the analysis of pro-inflammatory cytokines, pro-inflammatory chemokines, and anti-inflammatory cytokines.

Pro-Inflammatory Cytokines	Pro-Inflammatory Chemokines	Anti-Inflammatory Cytokines
GM-CSF	Eotaxin-1 (CCL11)	IL-1 Ra
IFN-γ	Eotaxin-3 (CCL26)	IL-4
IL-2*	IP-10 (CXCL10)	IL-10
IL-3	MCP-1 (CCL2)	IL-12/IL-23p40***
IL-5	MCP-4 (CCL13)	IL-13
IL-7	MDC (CCL22)	IL-22***
IL-8	MIP-1α (CCL3)	IL-27***
IL-9*	MIP-1β (CCL4)	
IL-12p70	MIP-3α (CCL20)	
IL-15	TARC (CCL17)	
IL-17A/F		
IL-17 Gen B.		
IL-17A, IL-17B, IL-17C, IL-17D		
IL-21*		
IL-23		
IL-31**		
TNF-β*		
TSLP		
VEGF-A		

CCL: C-C motif chemokine ligand; CXCL: C-X-C motif chemokine ligand; GM-CSF: Granulocyte-macrophage colony-stimulating factor; IFN: Interferon; IP: Interferon gamma-induced protein; MCP: Monocyte chemoattractant protein; MDC: Macrophage-derived chemokine; MIP: Macrophage inflammatory proteins; Ra = Receptor antagonist; TARC: Thymus and activation-regulated chemokine; TNF: Tumor necrosis factor; TSLP: Thymic stromal lymphopoietin; VEGF: Vascular endothelial growth factor; Ra = Receptor antagonist.

*Mainly pro-inflammatory but may also act as anti-inflammatory mediators.

**Not strictly a pro-inflammatory cytokine, but rather an immunoregulatory factor that induces pro-inflammatory cytokines.

***Mainly anti-inflammatory cytokine but may also act as pro-inflammatory mediators.

Supplemental Table S2. Effect of carrot juice intake on pro- and inflammatory biomarkers analyzed in an ex *vivo assay* using whole blood from healthy donors with and without addition of LPS. Time of blood sampling 0 h is before consumption of carrot juice and 1 h is after consumption of carrot juice (peak concentration of FaOH in the circulation [Jakobsen *et al. J. Chromatogr. B Analyt. Technol. Biomed. Life Sci.* **2022**, *1210*, 123440]). NSE = no significant effect. Mean concentrations (pg/mL) of biomarkers measured in the plasma sample are listed for the study (n = 14).

**Biomarker**	**Plasma from Whole Blood Before and After Intake of Carrot Juice**			**Plasma from LPS (10 μg/mL, 24 h)-Stimulated Whole Blood Before and After Intake of Carrot Juice**		
**Time of Sampling**	**0 h**	**1 h**		**0 h**	**1 h**	
	**Mean (pg/mL)**	**Mean (pg/mL)**	***p*-Value**	**Mean (pg/mL)**	**Mean (pg/mL)**	***p*-Value**
**Pro-Inflammatory Cytokines**						
GM-CSF	0.39	0.47	NSE	205.7	160.2	NSE
IFN-γ	26.5	26.9	NSE	252.7	337.4	0.0166
IL-1α[ǂ]	3108	3275	NSE	494226	410114	0.0419
IL-1β[ǂ]	70.4	120.5	NSE	4260	4268	NSE
IL-2*	5.2	6.5	NSE	49.3	139.3	NSE
IL-3	43.2	41.3	NSE	139.8	144.1	NSE
IL-5	1.2	1.0	NSE	5.7	4.9	NSE
IL-6[ǂ]	358.1	947.4	NSE	5431	5422	NSE
IL-7	4.7	4.8	NSE	14.2	39.0	NSE
IL-8	275.1	274.0	NSE	6187	6217	NSE
IL-9*	1.5	1.6	NSE	2.8	3.7	NSE
IL-12p70	7.0	12.6	NSE	58.9	65.1	NSE
IL-15	1.5	1.8	0.0105	2.0	2.2	NSE
IL-16[ǂ]	2339	2236	NSE	3912	2908	0.0085
IL-17A/F	4.0	4.0	NSE	11.5	12.9	NSE
IL-17A GenB	7.7	8.7	NSE	7.7	9.0	NSE
IL-17A	7.3	7.2	NSE	96.6	149.9	0.0023
IL-17B	6.3	7.2	NSE	15.1	19.2	0.0134
IL-17C	7.6	7.7	NSE	15.4	17.5	NSE
IL-17D	39.4	45.3	NSE	48.9	62.3	0.0085
IL-21	1.6	0.71	NSE	0.79	1.0	NSE
IL-23	2.5	4.5	NSE	135.9	207.9	0.0002
IL-31**	0.66	0.70	NSE	0.91	0.93	NSE
TNF α[ǂ]	17.9	17.9	NSE	8099	8479	NSE
TNF-β*	0.55	0.54	NSE	2.2	2.4	NSE
TSLP	3.2	2.4	NSE	23.3	23.2	NSE
VEGF-A	757.3	655.6	NSE	970.7	844.2	NSE
**Pro-Inflammatory Chemokines**						
Eotaxin-1 (CCL11)	1084	1040	NSE	1298	1245	NSE
Eotaxin-3 (CCL26)	119.0	108.8	NSE	222.2	188.0	NSE
IP-10 (CXCL10)	538.4	558.4	NSE	3105	3407	NSE
MCP-1 (CCL2)	5052	3296	NSE	4783	3759	NSE
MCP-4 (CCL13)	356.1	334.0	NSE	504.0	480.5	NSE
MDC (CCL22)	1041	997.7	NSE	1317	1284	NSE
MIP-1α (CCL3)	297.8	1179	NSE	11459	11448	NSE
MIP-1β (CCL4)	2123	2804	NSE	9752	9775	NSE
MIP-3α (CCL20)	8479	8773	NSE	14782	15532	NSE
TARC (CCL17)	162.9	147.4	NSE	203.3	183.9	NSE
**Anti-Inflammatory Cytokines**						
IL-1Ra	3305	3111	NSE	16921	17419	NSE
IL-4	4.3	6.9	NSE	43.0	40.3	NSE
IL-10	97.7	223.5	NSE	4268	3948	NSE

IL-12/IL-23p40***	133.3	141.3	NSE	3011	4131	0.0245
IL-13	35.4	34.8	NSE	101.7	96.7	NSE
IL-22***	4.4	4.9	NSE	8.0	11.2	0.0215
IL-27***	278.8	295.6	NSE	1913	2213	NSE

CCL: C-C motif chemokine ligand; CXCL: C-X-C motif chemokine ligand; GM-CSF: Granulocyte-macrophage colony-stimulating factor; IFN: Interferon; IP: Interferon gamma-induced protein; MCP: Monocyte chemoattractant protein; MDC: Macrophage-derived chemokine; MIP: Macrophage inflammatory proteins; Ra = Receptor antagonist; TARC: Thymus and activation-regulated chemokine; TNF: Tumor necrosis factor; TSLP: Thymic stromal lymphopoietin; VEGF: Vascular endothelial growth factor; Ra = Receptor antagonist.

ǂResults have been previously published (Deding *et al. Nutrients* **2023**, *15*, 632).

*Mainly pro-inflammatory but may also act as anti-inflammatory mediators.

**Not strictly a pro-inflammatory cytokine, but rather an immunoregulatory factor that induces pro-inflammatory cytokines.

***Mainly anti-inflammatory but may also act as pro-inflammatory mediators.
